# Amyloidosis of the Breast with Multicentric DCIS and Pleomorphic Invasive Lobular Carcinoma in a Patient with Underlying Extranodal Castleman's Disease

**DOI:** 10.1155/2013/190856

**Published:** 2013-02-04

**Authors:** David Chiang, Michael Lee, Pauline Germaine, Lydia Liao

**Affiliations:** Department of Radiology, Cooper University Hospital, Camden, NJ 08103, USA

## Abstract

We present an interesting case of focal amyloidosis of the left breast which was intermixed with ductal carcinoma in situ (DCIS). On subsequent staging bilateral breast magnetic resonance imaging (MRI), the patient was found to have an additional suspicious enhancing mass with spiculated borders within the left breast. This mass was biopsy proven to represent pleomorphic invasive lobular carcinoma. A pulmonary nodule within the lingula was also noted on the staging bilateral breast MRI and was biopsy proven to represent extranodal Castleman's disease. Therefore, it is believed that our patient had secondary amyloidosis due to Castleman's disease.

## 1. Introduction

Amyloidosis is caused by the deposition of insoluble beta-pleated fibrillar proteins throughout various tissues of the body [1]. In general, amyloid has an affinity for adipose tissue, making the breasts an optimal target [2]. Amyloid protein demonstrates a characteristic apple-green birefringence under polarized light after Congo red stain [3]. Although FNA may be helpful in the preliminary diagnosis of breast amyloidosis, histologic diagnosis from either core or excisional biopsy is required [3].

Amyloid deposits can distribute in a periductal, perivasculature or intralobular pattern [1]. They can result in a foreign body like reaction with infiltration of multinucleated giant cells. The multinucleated giant cells aid in the degradation of the amyloid deposits by releasing proteases [1]. In addition, amyloid deposits also can have an affinity for calcium, resulting in a mammographic appearance similar to DCIS or fibrocystic change [1]. It may also mimic inflammatory breast carcinoma with diffuse increased breast density with skin thickening [2]. Therefore, amyloidosis can have a wide range of mammographic appearances including benign or suspicious appearing masses and calcifications [2]. 

## 2. Case

**Figure 1:**
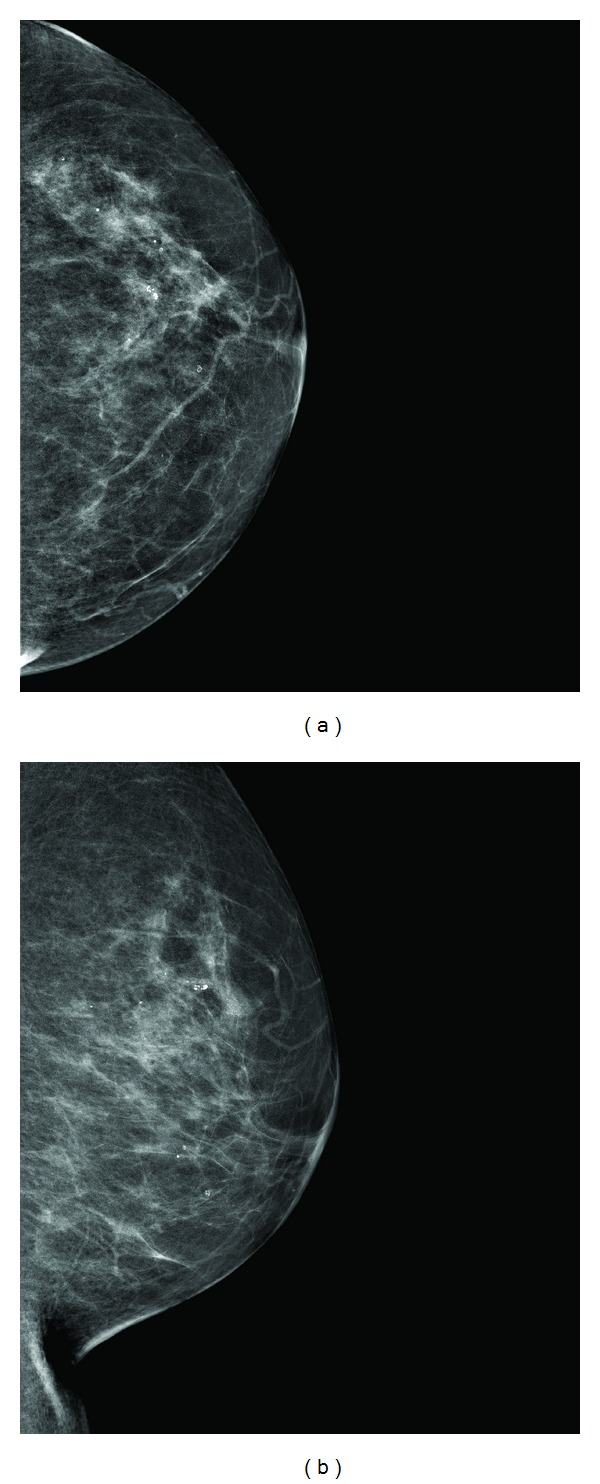
Craniocaudal (CC) and mediolateral oblique (MLO) projections of the left breast demonstrate suspicious pleomorphic calcifications in a segmental distribution within the superior-medial quadrant, spanning approximately 2.3 cm. These calcifications were biopsy proven to represent amyloidosis and DCIS.

Our patient is a 72-year-old female with no significant past medical history who presented to our institution for stereotactic vacuum assisted needle core biopsy of an already known suspicious 2.3 cm cluster of microcalcifications within the 11 o'clock region of the left breast (Figures 1(a) and 1(b)). No other suspicious mammographic abnormalities were noted in the left breast. Stereotactic biopsy demonstrated periductal and perivascular amyloid deposits, with the calcifications being related to amyloid deposition. There was no evidence of malignancy or atypia. However, due to the highly suspicious mammographic appearance, the pathology was felt to be discordant and patient with given a BIRADs 4 with recommendation for surgical excisional biopsy. WC instead underwent a repeat stereotactic vacuum assisted needle core biopsy of the same cluster of microcalcifications, which demonstrated DCIS and foci of atypical ductal hyperplasia; it should be noted that the repeat needle core biopsy was negative for amyloid. With the new diagnosis of DCIS, the patient underwent a staging bilateral breast MRI.

**Figure 2:**
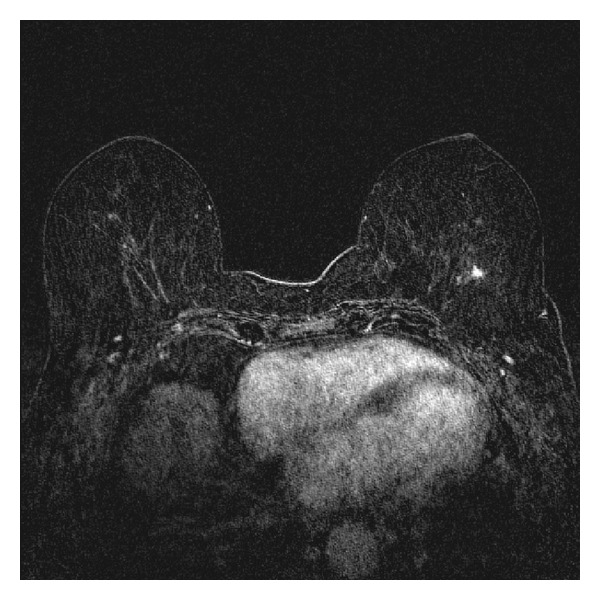
Subtraction MRI demonstrates a 0.9 cm enhancing mass with spiculated borders within the posterior left breast with no involvement of the left chest wall. This posterior mass was not appreciated on mammography. Under MRI guidance, this mass was biopsy proven to represent invasive pleomorphic lobular carcinoma.

**Figure 3:**
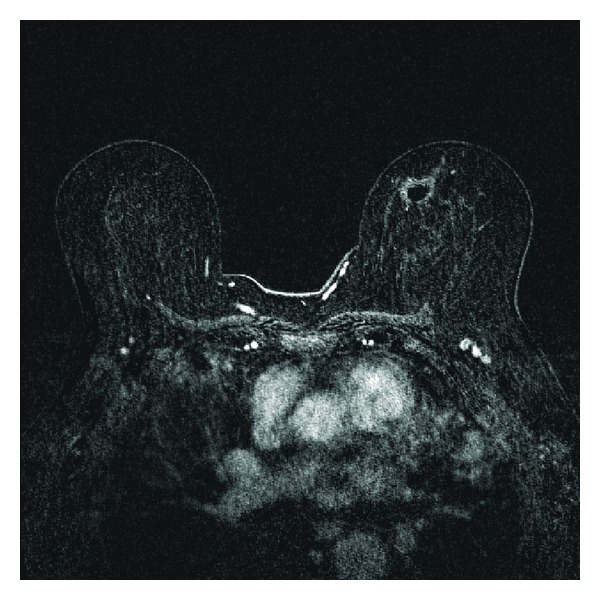
Subtraction MRI demonstrates a 2.1 cm × 1.2 cm enhancing mass within the lingula, biopsy proven to represent extranodal Castleman's disease. Incidental note is made of a postbiopsy hematoma/seroma within the medial left breast from stereotactic biopsy of the pleomorphic calcifications demonstrated in Figures 1(a) and 1(b).

 In addition to the known focus of DCIS within the 11 o'clock region of the left breast, a 0.9 cm avidly enhancing spiculated mass was also seen within the posterior 3 o'clock region of the left breast (Figure 2). No abnormalities were visualized within the right breast. However, the MRI also demonstrated a 2.1 cm × 1.2 cm nodule within the lingula of the left lung (Figure 3). Because the left breast 0.9 cm spiculated mass was not visualized on mammography, the patient then underwent an MRI guided biopsy of this second lesion. The pathology revealed a coexistent invasive pleomorphic lobular carcinoma.

 The patient underwent total left mastectomy, as breast conservation therapy was not plausible secondary to the multicentricity of the patient's cancer. Final surgical pathology demonstrated invasive pleomorphic lobular carcinoma and lobular carcinoma in situ at the 2-3 o'clock position. DCIS, intermediate nuclear grade, and solid and cribriform subtypes were demonstrated at the previous biopsy site around the 11 o'clock position. The sentinel lymph node was negative for metastatic carcinoma. Following the total left mastectomy, the patient also underwent a wedge resection of the lingula nodule. Pathology of the nodule demonstrated extranodal Castleman's disease of the plasma cell variant. Of note, there was no evidence of amyloid within this lingula nodule.

## 3. Discussion

Breast amyloidosis typically presents in women from 43 to 86 years of age, with only recent case reports describing their coexistence with breast cancers [1, 2]. Patients with breast amyloidosis may be clinically asymptomatic or present with palpable firm lesions, generalized tenderness, or peau d'orange skin findings [1]. Our patient presented with a nontender, palpable lump at around the 11 o'clock position of the left breast. She had no other clinical symptoms or radiographic findings to suggest multiorgan involvement of systemic amyloidosis. Although amyloidosis presented mammographically in our patient as a suspicious cluster of pleomorphic microcalcifications, it is difficult to determine whether this was solely due to amyloid because it was intermixed with DCIS.

 Previous case reports have described amyloidosis as distinct lesions or as intermixed with breast cancers. Reported coexisting breast cancers have included tubular carcinoma, invasive ductal carcinoma with extensive intraductal components, and invasive lobular carcinoma [4]. Our case is unique in that the patient presented with both focal amyloidosis intermixed with DCIS in addition to a separate focus of invasive pleomorphic lobular carcinoma within the same breast. In addition, our case is also unique in that the patient's localized breast amyloidosis was secondary to underlying extranodal Castleman's disease within the lingula. 

 Amyloidosis can be classified as either primary or secondary, with most cases of breast amyloidosis being classified as the later [4]. Secondary amyloidosis is characterized by the deposition of the acute phase protein, serum amyloid A (SAA) [5]. While primary amyloidosis is considered an idiopathic process, secondary amyloidosis is caused by an underlying chronic inflammatory disease such as inflammatory bowel disease, rheumatoid arthritis, or tuberculosis [4, 5]. In our patient, underlying extranodal Castleman's disease within the lingula was the underlying inflammatory process. Castleman's disease is an atypical lymphoproliferative disorder noted for giant lymph node hyperplasia [6]. Its association with amyloidosis is believed to occur secondary to the production of IL-6 by its hyperplastic lymph nodes. IL-6 results in the production of acute phase proteins in the liver, including SAA [6]. Complete surgical excision is considered curative for localized unicentric Castleman's disease [6].
